# Investigating the Association between Educational Attainment and Allostatic Load with Risk of Cancer Mortality among African American Women

**DOI:** 10.21203/rs.3.rs-2644466/v1

**Published:** 2023-03-27

**Authors:** Cynthia Li, Sydney Elizabeth Andrzejak, Samantha R. Jones, Brittany Marie Williams, Justin Xavier Moore

**Affiliations:** Augusta University; Augusta University; Augusta University; University of Vermont; Augusta University

**Keywords:** cancer, life-course, cumulative stress, psychosocial stress, race, disparities, allostatic load

## Abstract

**Background:**

African American (AA) women navigate the world with multiple intersecting marginalized identities. Accordingly, AA women have higher cumulative stress burden or allostatic load (AL) compared to other women. AL correlates with poorer health outcomes and increased risk of cancer death. However, research indicates AA women with a college degree or higher have lower AL than AA women with less than a high school diploma. We examined whether educational attainment differences and AL status in AA women are associated with long-term risk of cancer mortality.

**Methods:**

We performed a retrospective analysis among 4,677 AA women respondents using National Health and Nutrition Examination Survey (NHANES) data from 1988 through 2010 with follow up data through December 31, 2019. We fit Cox proportional hazards models to estimate adjusted hazard ratios (aHRs) of cancer death between educational attainment/AL (adjusted for age, sociodemographic, and health factors).

**Results:**

AA women with less than a high school diploma living with high AL had nearly a 3-fold increased risk (unadjusted HR: 2.98; 95%C CI: 1.24–7.15) of cancer death compared to AA college graduates living with low AL. However, after adjusting for age, the increased risk of cancer death in those with less than a high school diploma and high AL attenuated (age-adjusted HR: 1.11; 95% CI: .45–2.74).

**Conclusions:**

Differences in educational attainment and AL in AA women were not associated with increased risk of cancer mortality when adjusted for age. Previous studies have shown that increased allostatic load is associated with increased risk of cancer death. However, for African American women, higher educational attainment does not modify the risk of cancer mortality. The benefits that may come along with higher education such as increased access to medical care and better medical literacy do not change the risk of cancer mortality in AA women.

## Introduction

Chronic stress has been linked to worse health outcomes for various diseases, from atherosclerosis and depression to hypertension(1, 2). The body undergoes physiologic changes to compensate for chronic stress. There is an increased production of corticosteroids and catecholamines, an increased inflammatory response, increased levels of oxidative stress, and DNA damage(3, 4). Dai et al. suggest that this cumulative effect and physiologic changes promote tumorigenesis and cancer development by suppressing immunity and enhancing inflammation(3).

Allostatic load (AL) measures the cumulative burden of chronic stress on physiological systems. Chronic stress can result from major life events, but more importantly, is the summative effect of ordinary, everyday stressors such as poor sleep, a lack of exercise, and a lack of access to healthy food(5). A cross-sectional study by Allen et al. found that “racial discrimination may be an important predictor of cumulative physiologic dysregulation.”(6) Experiences of racial discrimination as a stressor have been correlated with worse physical and mental health outcomes(7–9). The “weathering” hypothesis by ***Geronimus et al***. suggested that systemic stress from cumulative socioeconomic disadvantage and political marginalization contribute greatly to the early and disproportionate health deterioration of African Americans (AA) (10).

Multiple studies have illuminated that Black men and women consistently have the highest allostatic load scores, and, thus consistently higher chronic stress, compared to those of the same age in other racial groups(10–12). Black women navigate this world with multiple, intersectional marginalized identities, meaning they are subject to multiple forms of layered discrimination by race, sex, class, and other social group categories. Black women consistently have higher allostatic loads compared to their Black male and White female counterparts(6, 13). A longitudinal study by ***Upchurch et al,*** observed that African American women reported higher levels of “discrimination, perceived stress, and hostility”, all of which will increase allostatic load(14). In a cross-sectional study, ***Moore et al*.** found that Black women had the highest age-adjusted mean allostatic load scores at the end of 30 years compared to others in their same age group(15). Considering that allostatic load is a measure of chronic stress, and that chronic stress has been associated with biological changes that promote tumorigenesis, it is worthwhile to understand the correlations between allostatic load in Black women and cancer mortality(3). Several studies have shown that African Americans generally have the highest cancer mortality burden(16, 17).

Furthermore, ***Williams et al*.** found that Black women with lower educational attainment had a higher allostatic load(18). Therefore, considering educational attainment’s impact on allostatic load is important in understanding the specific effects of chronic stress on health outcomes. AL and chronic stress are directly associated with worse health outcomes in multiple disease states, particularly cancer(3, 16, 17). The present study explored the relationship between educational attainment levels and allostatic load in AA women and their association with long-term risk of cancer mortality.

## Methods

### Study Design and Participants:

**Figure 1:**
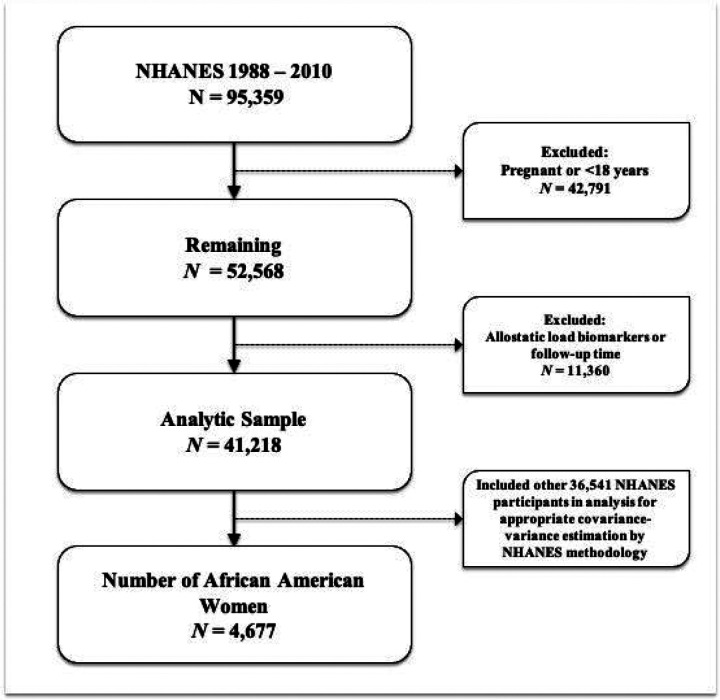
Flowchart of exclusion criteria and final study population of NHANES participants.

We performed a retrospective cohort analysis using data from the National Health and Nutrition Examination Survey (NHANES), a representative sample of non-institutionalized US residents linked with the National Center for Health Statistics (NCHS) 2019 National Death Index (NDI) file. The NHANES program oversamples those aged 60 and older, Latinx and non-Hispanic (NH)-Black individuals, and weighted analysis generates generalizable estimates (19). The weighted sample of NHANES is comparative to non-institutionalized United States (U.S.) population (20). Using NHANES survey data from years 1988 through 2010 linked with NDI data (follow-up data through December 31, 2019) we examined the association between the intersectionality of educational attainment and allostatic load with risk of cancer mortality. The NHANES survey includes information on sociodemographics, clinical measurements, and health-related questionnaires. NHANES participants with data on biomarkers were used in this analysis. We performed analysis among NHANES participants with data on biomarkers and within a fasting subsample (N = 95,359). Patients were excluded if they reported current pregnancy or were less than 18 years of age (N = 42,791), were missing AL biomarkers or not linked via NDI (N= 11,360). This resulted in a final analytic sample of NHANES participants aged 18 and older, corresponding to a total of 41,218 participants over a 22- year study period, of which 4,677 identified as African American women (Figure 1). We completed analyses using domain statements to account for appropriate estimations of covariance-variance structures using specific strata, cluster, and weighting procedures as specified by NHANES methodology. We created a domain variable based on the intersectionality of race/ethnicity with sex specified at survey, and thus we had an eight level variable containing: (1) African American (AA) men, (2) AA women, (3) NH-White men, (4) NH-White women, (5) Hispanic men, (6) Hispanic women, (7) Other/mixed race men, and (8) Other/mixed race women. Mortality status or vital status for participants was determined through NHANES-NDI linked file.

### Ethical Statement:

The Institutional Review Boards considered this study exempt from review because of the use of secondary, publicly available, and de-identified data.

### Independent Variable and Other Variables of Interest.

This study mirrored methods of our investigative team’s prior work, ***Williams et al*** (2022), to determine our independent variables and other variables of interest(18). We examined educational attainment as the primary independent variable of interest which was determined from the NHANES question “What is the highest grade or level of school you completed or highest degree received?”. We then categorized educational attainment into a four-level variable based on participants’ that completed (1) less than an high school (HS) education; (2) high school graduate, general education development test (GED), or equivalent; (3) some college; and (4) college graduate or above (18). Due to the NHANES data collection, we could not differentiate by specific degree types (e.g., MD, PhD, MSN).

We included other variables as covariates based on their consideration as potential confounders, or their possible effect on education, cumulative stress, and cancer outcomes based on prior studies. These variables included NHANES baseline survey completion period (e.g., 1988–1991 through 2009–2010), family poverty-to-income ratio (PIR), current smoking status, any self-reported history of cancer, congestive heart failure, and ever heart attack. PIR was calculated as the ratio of total family income to poverty threshold values by NHANES investigators (18). Participants that reported no income were given a zero value for PIR (18). PIR values greater than 1 are above the poverty level, and values near 5 are considered very high income, while PIR values less than 1 are considered below the official poverty line (18). Participants who had smoked at least 100 cigarettes in their lifetime and who were currently smoking during survey administration were categorized as current smokers (18). We determined self-reported diagnoses by doctor for cancer, congestive heart failure, or heart attack from NHANES questionnaires on whether “...a doctor or other health professional ever told you that you had ... (cancer, angina, congestive heart failure (CHF), or heart attack” (18).

### Allostatic Load Definition.

AL has been defined using varying components, although most incorporate biomarker measures from three different categories, including physiologic functioning, which incorporates cardiovascular, metabolic, and immune systems(21). While there is no consensus definition, we decided to define AL using the *Geronimus et al*. (2006) and *Moore et al*. (2021) taxonomies (10, 15). AL components included body mass index (BMI), diastolic blood pressure (DBP), glycohemoglobin (hemoglobin A1c), systolic blood pressure (SBP), total cholesterol, serum triglycerides, serum albumin, serum creatinine, and C-reactive protein (CRP). We considered sex as a biological variable according to National Institutes of Health guidelines regarding human subjects research(22) (23). To determine the high-risk thresholds for each AL component, we examined the sex reported at survey-specific distributions of each component among the entire study sample with complete biomarker data. High-risk thresholds were determined by either being above the 75^th^ percentile for BMI, CRP DBP, glycated hemoglobin, SBP total cholesterol, serum triglycerides, and serum creatinine(24, 25) or below the 25^th^ percentile for serum albumin. Therefore, each NHANES participant was scored as either 1 (high-risk) or 0 (low-risk) based on sex at baseline survey-specific cutoffs for each component. Total AL score was calculated by summing the individual components, ranging from 0 to 9. Participants were further categorized with AL scores greater or equal to 3 as having high AL (21, 26).

### Allostatic Load and Educational Attainment.

After categorizing NHANES participants based on the distribution of AL components and their self-reported educational attainment, we created a variable examining the intersection of AL and educational attainment. This variable was categorized into eight levels; (1) college graduate or more living with low AL (n = 256), (2) college graduate or more living with high AL (n = 258), (3) some college with low AL (n = 524), (4) some college with high AL (n = 598), (5) HS diploma or equivalent with low AL (n = 662), (6) HS diploma or equivalent with high AL (n = 849), (7) less than HS with low AL (n = 473), and (8) less than HS with high AL (n = 1,044).

### Primary Outcome of Interest, Cancer DeathOur

Deaths attributed to malignant neoplasms (ICD-10 019–043) were included as cancer-related deaths. Our primary outcome of interest was time to cancer-related death. Follow-up data for this analysis was available through December 31, 2019 based on NDI-NHANES publicly available linkages. The primary determination of mortality for eligible NHANES participants is based upon matching survey records to the NDI although additional redundant sources are also incorporated, including the Social Security Administration, the Centers for Medicare and Medicaid Services, data collection, NCHS’ follow-up surveys (e.g., NHEFS), and ascertainment of death certificates.

### Statistical Analysis:

Primary analyses were conducted using NHANES-generated sampling statistical strata, clusters, and weights as designated and described in detail within the NHANES methodology handbook(27). NHANES only measures biomarkers among a random sample of participants each survey period, and in turn creates subsample weights to account for the probability of being selected into the subsample component and additional non-response bias. Categorical variables were presented as weighted row percentages and continuous variables as mean and associated 95% confidence intervals. Mean survival times were estimated using the product-limit method of the KaplanMeier survival estimator. Proportionality assumption was assessed for our primary variable of interest (education attainment by allostatic load status) by examining the proportion of 1000 simulations that contain a maximum cumulative martingale residual larger than the observed maximum cumulative residuals using the SAS procedure ‘supremum test’. None of our exposure levels had p values that were statistically significant (p value <0.05), and therefore none of our residuals were larger than expected and we did not reject proportional hazards assumptions. Relative rates of cancer death by groups of educational attainment/allostatic load were estimated by fitting survey-weighted Cox proportional hazards models with time-to-cancer death as the endpoint^16^. Individuals were censored at the time of their event, death, or end of follow-up (December 31, 2019). Models were sequentially adjusted first for age, then with age, poverty to income ratio, and smoking status.

Multiplicative interactions of AL and educational attainment were examined by introducing an interaction term within our model and presenting the corresponding p-value for this association. P-values <0.05 were considered statistically significant. Additionally, we conducted all the time-to-cancer death event survival analyses by allostatic load status (high versus low allostatic load), stratified by educational attainment. Estimates were presented from our survey-weighted Cox proportional hazard models as hazard ratios (HRs) and associated 95% confidence intervals (CIs). All statistical analyses were performed using SAS (version 9.4, SAS Institute, Inc., Cary, North Carolina, USA).

## Results

Among 4,677 (an estimated 9,381,049) NH-Black women, the average age of participants was 42.71 years (Standard Error (SE) = 0.33) and the median follow-up time was 15.90 years (Q1-Q3 = 11.48 – 21.88) (Table 1). NH-Black women with less than high school education attainment living with high allostatic load were on average older (55.5 years, SE = 0.7) than all other groups while those with high school diploma or equivalent education attainment living with low allostatic load were on average younger (32.8 years, SE = −.6) than all other groups (p value <0.01). Participants with college graduate or more educational attainment living with high allostatic load (mean PIR = 3.6, SE = 0.1) and living with low allostatic load (mean PIR = 3.5, SE = 0.1) had much higher income than all other groups (p value <0.01). The group of participants with highest rate of current smoker status was that of less than high school educational attainment and living with high allostatic load (N=267, SE 28.2). Generally, participants living with high allostatic load (4.9%, 5.0%, 5.1 %, 5.3%) were more likely to have a history of cancer compared to those living with low allostatic load (1.9%, 2.8%, 2.6%, 2.5%), among those with less than high school, high school or equivalent, some college, and college graduates or more, respectively (p value <0.01). Those identified as college graduates or more with high allostatic load had the highest prevalence of obesity-related cancer (3.2%). Participants with less than high school attainment and high allostatic load had the highest prevalence of congestive heart failure (6.2%) and history of heart attack (5.1%).

Participants with education attainment of less than high school living with high allostatic load had nearly a 3-fold increased risk (unadjusted HR: 2.98; 95% CI: 1.24–7.15) of dying from cancer compared to college graduates living with low allostatic load (Table 2) However, after adjusting for age, we observed that the association between participants having less than high school educational attainment living with high allostatic load and risk of cancer death attenuated (age-adjusted HR: 1.11; 95% CI: 0.45–2.74). We observed no other statistically significant associations between educational attainment groups and allostatic load status with risk of cancer death. When stratified by educational attainment, we observed that participants with less than a high school degree with high allostatic load had an approximately 3-fold increase in risk of cancer death when compared to those with low allostatic load (unadjusted HR: 3.28; 95% CI: 1.88–5.73). Similarly, among those with a high school diploma or equivalent, there was over a 3.5-fold increase in risk of cancer death in those with high allostatic load compared to those with a low allostatic load (unadjusted HR: 3.61; 95% CI: 1.92–6.76). However, when adjusted for age, the risk of cancer death was attenuated for both the less than high school degree and high school diploma or equivalent groups (<HS age-adjusted HR: 1.41 ; CI: 0.7402.7; HS diploma or equivalent age-adjusted HR: 1.76, CI: 0.94–3.35). Lastly, among participants with college graduate or more, those with high allostatic load had a nearly 2-fold increase in cancer death compared to those with low allostatic load (unadjusted HR: 1.94; 95% CI: 1.39–2.71). This effect, too, was similarly attenuated when adjusted for age (age-adjusted HR: 0.82; 95% CI: 0.25–2.72). No other statistically significant associations between educational attainment groups and allostatic load status with risk of cancer death were found when stratified by educational attainment. We observed similar effect measures for educational attainment with allostatic load when conducting analysis with Poisson regression estimating relative risks of cancer death (Supplemental Table 1).

## Discussion

In this study, we examined the relationship between educational attainment and allostatic load in AA women and its association with a long-term risk of cancer mortality. AA women with education attainment of less than high school diploma and living with high allostatic load had nearly a 3-fold increased risk of dying from cancer when compared to college graduates living with low allostatic load. However, after adjusting for age, the association between participants having less than high school educational attainment and living with high AL attenuated the risk of cancer death. Even when stratified by educational attainment status, we observed that the relationship between AL and cancer mortality reduced after adjusting for confounders.

Reductions in AL that may be obtained with higher education, including increased access to healthcare and better understanding of the healthcare system, did not improve cancer mortality. This means many of the perceived health benefits of a college education do not result in improved health outcomes for AA women with cancer. Previous studies have found that socioeconomic factors alone are insufficient to explain the effect of race on cancer outcomes among Black women(16, 17). Socioeconomic variables in conjunction with cultural beliefs and attitudes, may largely account for sustained disparities in cancer mortality among Black women(28, 29). Further, increased health literacy for Black women with higher education, differences in tumor phenotype, inherited predispositions, comorbidities, and discrimination and bias experienced by Black women may also account for sustained cancer mortality rates(30, 31).

The findings in Moore et. al.’s study found that despite living in closer proximity to available healthcare services, increased odds of late-stage diagnosis, no receipt of treatment, and risk of breast cancer death were sustained for NH-Black women living in urban environments compared to rural NH-Black women(32). One explanation to our findings may be a relative homogeneity in AL in AA women. Our standardization of AL was based on relative data from all races from the NHANES survey. Therefore, it may be possible that differences in high and low AL in AA women were minimal, and therefore, it is difficult to establish a difference in cancer mortality between the two groups.

Previously, Williams et al. determined that AA women with a baccalaureate degree or higher had lower AL(18). This finding further confirmed existing data suggesting that higher education was a social determinant of health(6, 33, 34). However, when looking specifically at AA women, AL and cancer mortality, we found that increased educational attainment and its relationship with AL did not improve cancer mortality. This lack of improvement raises questions about the efficacy of education in mitigating poorer health outcomes, with specific attention to cancer survival and death. Further, Moore et al. observed that high AL was associated with an increased risk of overall cancer death(15). Our results mirrored those of Moore et al., finding that AA women with high AL were more likely to have a history of cancer compared to those living with low AL. However, there was no statistically significant difference in cancer mortality between educational level and high or low AL. Accordingly, future researchers should examine whether the racial weathering associated with living while Black in the U.S. wholly obscures the possibilities of educational attainment mitigating cancer death outcomes. Several studies have shown that AA people generally have the highest cancer mortality burden(16, 17). With our findings, it does not appear that increased education changes the rate of cancer mortality for AA women. The added benefits of higher education, including possible increased income and decreased chronic stress, did not lead to decreased cancer mortality. There may also be other factors at play. It is possible that AA women’s lived experiences with the healthcare system may play a role in attenuating any benefits of higher education and AL may have on cancer mortality. In addition, a study by Hudson et al. found that the process of upward social mobility may not lend itself to improved health outcomes for AA men and women in the same way that upwardly mobile NH White men and women experience(35). Meaning, upward mobility may instead be associated with greater health burdens for AA compared to their White counterparts. Indeed, upward mobility may increase experiences with racism and decrease social support. Those with higher educational attainment may attenuate any improvements in cancer mortality associated with higher education in other races due to the stress of negotiating these classed spaces while Black. Future scholars may wish to examine this and other physician-patient factors and their possible role in cancer mortality.

The results from this study should be contextualized by the strengths and limitations of our data. One limitation to our study includes a subsample of non-Hispanic Black women. Further, allostatic load and baseline exposure variables were attained at a single point in time and not re-assessed. We cannot elucidate life course factors and events between exposure and outcomes. There may be other factors in play between the time of the survey and interview and the time they passed from cancer. Another limitation is in the initial collection of the educational data, wherein there was limited disaggregation for those with at least a baccalaureate degree or higher. Our inability to analyze whether differences at the baccalaureate versus the postgraduate levels forced us to make incomplete inferences about educational attainment, AL, and cancer mortality. However, some strengths of our study include using a nationally representative sample. This allows us to generalize our findings better. Further, this is one of the first studies to look at AL and the risk of cancer death, specifically among AA women.

## Conclusion

Previous studies have shown that increased allostatic load is associated with increased risk of cancer death. However, for African American women, higher educational attainment does not modify the risk of cancer mortality. Accordingly, educational attainment and AL differences in AA women were not associated with increased risk of cancer mortality once adjusted for age. Our findings reveal the benefits associated with higher education, such as increased access to medical care and better medical literacy do not change AA women’s risk of cancer mortality. Further research is needed to better understand the factors affecting AA women’s lives that may contribute to higher rates of cancer mortality and mediating differences in AL.

## Figures and Tables

**Table 1:** Socio-demographic characteristics, personal health, and medical conditions by allostatic load and educational attainment, National Health Examination Survey (NHANES) study period. Among 4,677 NHANES survey participants (an estimated 9,381,049 non-institutionalized US non-Hispanic Black women) years 1988 through 2010 and follow up through December 31, 2019.

	Living with High Allostatic Load^a^				Living with Low Allostatic Load			
	< High School	High School or Equivalent	Some College	College Graduate or more	< High School	High School or Equivalent	Some College	College Graduate or more
**Unweighted sample size** ^b^	1044	849	598	258	473	662	524	256
**Weighted sample size** ^b^	1,689,042	1,526,515	1,360,399	561,849	941,705	1,242,292	1,364,856	690,186
**Presented as % or Mean with SE** ^c^								
**Allostatic load total score** ^d^	4.5 (0.04)	4.2 (0.05)	4.2 (0.06)	4.4 (0.09)	1.1 (0.05)	1.2 (0.03)	1.1 (0.04)	1.2 (0.05)
**Mean age in years**	55.5 (0.7)	45.7 (0.6)	45.3 (0.8)	47.7 (1.0)	36.1 (0.7)	32.8 (0.6)	35.0 (0.6)	37.8 (0.7)
**Age Group**								
18 – 29	81 (7.1)	154 (16.7)	95 (16.6)	27 (11.4)	226 (40.3)	377 (47.9)	217 (40.1)	58 (24.0)
30 – 39	118 (12.8)	170 (20.1)	115 (20.2)	47 (17.5)	99 (26.8)	148 (26.8)	138 (28.7)	90 (35.4)
40 – 49	149 (18.0)	192 (24.7)	143 (27.2)	58 (26.1)	52 (14.4)	72 (14.4)	99 (19.9)	64 (25.8)
50 – 59	167 (18.5)	120 (17.0)	91 (18.3)	56 (26.1)	34 (9.3)	32 (6.5)	31 (6.5)	28 (11.4)
60 – 69	253 (20.2)	137 (13.4)	106 (12.1)	42 (10.9)	26 (3.6)	19 (2.4)	22 (2.5)	13 (2.7)
70+	276 (23.6)	76 (8.1)	48 (5.7)	28 (8.1)	36 (5.6)	14 (2.1)	17 (2.3)	3 (0.7)
**Time Period** ^e^								
1988–1991	245 (21.0)	182 (21.6)	82 (9.9)	40 (4.3)	82 (8.4)	146 (17.2)	88 (12.1)	32 (4.8)
1991–1994	342 (24.8)	347 (27.6)	143 (11.1)	72 (6.4)	77 (5.2)	171 (13.3)	90 (7.8)	42 (3.6)
1999–2000	79 (18.5)	39 (10.4)	34 (11.2)	13 (3.5)	68 (16.7)	47 (12.9)	48 (17.0)	26 (8.9)
2001–2002	63 (13.9)	46 (12.4)	53 (15.7)	21 (5.9)	67 (16.0)	57 (13.2)	49 (15.5)	21 (7.3)
2003–2004	69 (17.0)	51 (12.8)	51 (15.3)	25 (6.9)	47 (10.6)	68 (12.9)	53 (16.6)	22 (7.7)
2005–2006	74 (15.0)	59 (12.0)	70 (17.5)	28 (7.2)	49 (9.0)	60 (9.3)	74 (21.1)	34 (8.8)
2007–2008	94 (15.5)	69 (12.9)	75 (15.4)	33 (7.5)	45 (9.5)	51 (10.7)	63 (15.4)	52 (13.0)
2009–2010	68 (14.0)	56 (12.4)	90 (20.5)	26 (6.1)	38 (9.5)	62 (15.0)	59 (15.4)	27 (7.1)
**Mean Family PIR**	1.5 (0.05)	1.9 (0.06)	2.4 (0.08)	3.6 (0.1)	1.4 (0.07)	1.7 (0.06)	2.3 (0.08)	3.5 (0.1)
**Current smoker status**	267 (28.2)	215 (26.8)	113 (17.4)	35 (13.1)	136 (34.6)	145 (25.4)	112 (20.7)	19 (6.4))
**Any cancer history** ^f^	53 (4.9)	38 (5.0)	35 (5.1)	14 (5.3)	8 (1.9)	14 (2.8)	12 (2.6)	6 (2.5)
**CHF**	64 (6.2)	25 (2.9)	20 (3.2)	5 (1.9)	9 (1.7)	3 (0.3)	3 (0.6)	1 (0.2)
**Ever Heart attack**	54 (5.1)	27 (2.8)	22 (3.4)	5 (1.8)	8 (1.7)	3 (0.3)	5 (0.9)	1 (0.5)

a High Allostatic load is defined as total Allostatic load score greater than or equal to 3 (presented as column percentages and standard errors).

b Unweighted sample size.

c Estimated using sampling weights from National Health and Nutrition Examination Survey (NHANES).

d Presented as unweighted column sample size (weighted percentage) or mean (standard error) for continuous variables.

e Presented as unweighted row sample size (weighted percentage)

f Defined as self-reported response to ever being diagnosed by a doctor or health professional of any cancer or malignancy.

**Table 2:** Survey weighted Cox proportional hazard models presented as Hazard Ratios (HR) and 95% Confidence Intervals (CI) for the association between educational attainment/allostatic load and risk of cancer death, among 4,677 (weighted ***N=***9,381,049) NHANES participants with 242 (weighted n = 394,768) cancer-related deaths.

	No. & (Weighted %) Cancer Deaths	Mean Survival Months (SE)	Hazard Ratio (HR) and 95% Confidence Interval (Cl)		
Educational Attainment and Allostatic Load Status			Unadjusted	Age Adjusted	Fully Adjusted
College graduate or more with low allostatic load	6 (2.7)	308.6 (1.9)	1.00 (Referent)	1.00 (Referent)	1.00 (Referent)
College graduate or more with high allostatic load	12 (4.4)	275.5 (2.0)	1.62 (0.49 – 5.30)	0.92 (0.28 – 3.02)	0.79 (0.24 – 2.61)
Some college with low allostatic load	17 (2.8)	246.2 (1.2)	1.02 (0.32 – 3.26)	1.19 (0.37 – 3.83)	1.00 (0.30 – 3.26)
Some college with high allostatic load	28 (3.4)	235.8 (1.2)	1.32 (0.48 – 3.66)	0.85 (0.30 – 2.41)	0.70 (0.24 – 2.01)
HS diploma or equiv. with low allostatic load	12 (1.7)	260.7 (0.7)	0.55 (0.21 – 1.45)	0.68 (0.26 – 1.76)	0.53 (0.21 – 1.35)
HS diploma or equiv. with high allostatic load	55 (6.1)	251.8 (1.3)	1.97 (0.75 – 5.17)	1.25 (0.49 – 3.18)	0.98 (0.38 – 2.49)
<HS with low allostatic load	15 (2.5)	290.7 (1.7)	0.90 (0.33 – 2.44)	0.87 (0.32 – 2.41)	0.63 (0.22 – 1.75)
<HS with high allostatic load	96 (7.5)	294.8 (2.1)	2.98 (1.24 – 7.15)	1.11 (0.45 – 2.74)	0.82 (0.33 – 2.04)
**Educational Attainment Stratified Results**					
**Among participants with <HS**					
Low allostatic load	15 (2.5)	290.7 (1.7)	1.00 (Referent)	1.00 (Referent)	1.00 (Referent)
High allostatic load	96 (7.5)	294.8 (2.1)	3.28 (1.88 – 5.73)	1.41 (0.74–2.7)	1.39 (0.73–2.66)
**Among participants with HS diploma or equiv.**					
Low allostatic load	12 (1.7)	260.7 (0.7)	1.00 (Referent)	1.00 (Referent)	1.00 (Referent)
High allostatic load	55 (6.1)	251.8 (1.3)	3.61 (1.92 – 6.76)	1.76 (0.94–3.35)	1.76 (0.93–3.33)
**Among participants with Some college**					
Low allostatic load	17 (2.8)	246.2 (1.2)	1.00 (Referent)	1.00 (Referent)	1.00 (Referent)
High allostatic load	28 (3.4)	235.8 (1.2)	1.30 (0.60 – 2.84)	0.66 (0.27–1.62)	0.66 (0.27–1.62)
**Among participants with college graduate or more**					
Low allostatic load	6 (2.7)	308.6 (1.9)	1.00 (Referent)	1.00 (Referent)	1.00 (Referent)
High allostatic load	12 (4.4)	275.5 (2.0)	1.94 (1.39 – 2.71)	0.82 (0.252.72)	0.75 (0.21–2.65)
p-value for interaction between education and allostatic load			<0.01	<0.01	<0.01

Percentages are weighted. Cox proportional hazard models are estimated using NHANES survey weighting.

Fully adjusted is for age, family poverty to income ratio, and current smoker status.

## Data Availability

The datasets generated or analyzed during the current study are available in the Centers for Disease Control and Prevention National Health and Nutrition Examination Survey (NHANES) repository, https://wwwn.cdc.gov/nchs/nhanes/

## Abbreviations

AA: African American
AL: Allostatic Load
NHANES: National Health and Nutrition Examination Survey
aHRs: adjusted hazard ratios
NCHS: National Center for Health Statistics
NDI: National Death Index
NH: non-Hispanic
U.S.: United States
HS: High School
GED: General Education Development test
PIR: Poverty to Income Ratio
CHF: congestive heart failure
BMI: body mass index
DBP: diastolic blood pressure
hemoglobin A1c: glycohemoglobin
SBP: systolic blood pressure
CRP: C-reactive protein
HR: hazard ratios
CIs: confidence intervals
